# Transient Acquired Hypoventilation Syndrome Secondary to Uncal Herniation Is Successfully Treated with Bilevel Noninvasive Positive Pressure Ventilation

**DOI:** 10.1155/2018/7013916

**Published:** 2018-12-17

**Authors:** Linle Hou, Jonathan Stoll, Lauren Pioppo, Jack Xu, Wajahat Khan

**Affiliations:** ^1^Department of Internal Medicine, Rutgers Robert Wood Johnson Medical School, New Brunswick, NJ, USA; ^2^Penn Medicine Princeton Health, Plainsboro, NJ, USA

## Abstract

**Background:**

To describe an unusual presentation of acquired hypoventilation syndrome treated successfully with noninvasive positive pressure ventilation.

**Case Presentation:**

We report a case report of a 48-year-old male who presented to the emergency room for recurrent syncope. He was found to have a ventricular colloid cyst causing uncal herniation. The patient was noted to be intermittently apneic and bradypnic. Transient hypoventilation was successfully treated with noninvasive positive pressure ventilation and the patient made a full neurological recovery following transcallosal resection of the colloid cyst. Subsequently, the hypoventilation resolved.

**Conclusion:**

With prompt surgical intervention, full neurological recovery is possible after cerebral uncal herniation. In rare circumstances, this can result in transient alveolar hypoventilation. Bilevel noninvasive positive pressure ventilation can be used to successfully manage the hypoventilation.

## 1. Background

Central hypoventilation syndrome (CHS) is characterized by the disruption of integration signals in ventilatory control system resulting in hypoventilation, hypoxemia, and hypercapnia. The congenital form of CHS is linked to genes involved in the neuronal development of autonomic nervous system [1]. Brainstem injury due to ischemia, inflammation, or trauma can result in acquired central hypoventilation syndrome (ACHS). Management of ACHS usually consists of supportive care and adequate ventilatory support [2]. The latter can be achieved with a variety of therapies, including invasive mechanical ventilation, negative pressure ventilation, noninvasive positive pressure ventilation (NIPPV), or nocturnal diaphragmatic pacing. Cerebral uncal herniation puts pressure on the brainstem and usually results in long-term ACHS. Transtentorial uncal herniation is often fatal. Those who do survive frequently suffer lifelong neurological sequelae. Transient ACHS due to uncal herniation is rare, and management strategies for hypoventilation are lacking in the literature. We report a case of transient ACHS due to uncal herniation, which was successfully treated with bilevel noninvasive positive pressure ventilation.

## 2. Case Presentation

**Figure 1:**
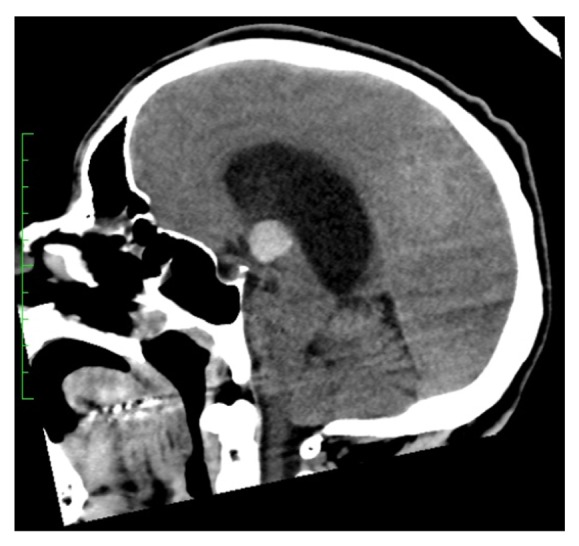
The marked lateral ventricle hydrocephalus secondary to a 16 x 15 x 15 mm colloid cyst within the third ventricle seen on CT scan at admission from the sagittal view.

**Figure 2:**
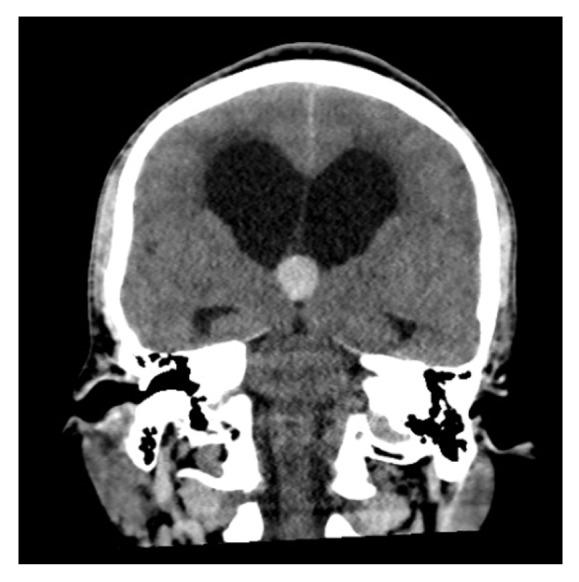
Similar marked hydrocephalus and colloid cyst seen from the coronal view.

A 48-year-old male presented to the emergency room for recurrent syncope. He became acutely unresponsive while in the emergency room. His pupils became fixed and dilated bilaterally. Emergent computed tomography (CT) of the head revealed a ventricular colloid cyst with hydrocephalus and uncal herniation as shown in Figures 1 and 2. Emergent bedside bilateral external ventricular drains were placed in the emergency room and the patient was subsequently intubated for airway protection. He was noted to be intermittently apneic and bradypnic on mechanical ventilation. The low minute ventilation persisted after liberation from mechanical ventilation. This was monitored with noninvasive end-tidal carbon dioxide detection and arterial blood gas sampling. The transient ACHS was managed with bilevel NIPPV intermittently, as needed for hypercapnia during wakefulness and continuously through sleep. The hypoventilation resolved after three to four days. Arterial blood gas sampling at that time revealed a PaCO_2_ of 35 mm Hg. NIPPV was discontinued and the patient made a full functional neurologic recovery. He underwent a successful transcallosal resection of the colloid cyst.

## 3. Discussion

Congenital central hypoventilation syndrome (CCHS) usually affects infants, and symptoms often become apparent shortly after birth. Hypoventilation is typically more severe during sleep than wakefulness [3]. Etiologies of CCHS can range from infection such as poliomyelitis, as well as autonomic dysfunction, demyelination, cervical spine trauma, anoxia, and ischemic etiologies [4–6]. Management of CCHS requires chronic ventilatory support with positive pressure ventilation via tracheostomy. In such cases, NIPPV is not an appropriate long-term treatment option.

ACHS, in contrast, usually results from brainstem lesions or insults affecting the medullary centers that control respiratory drive [3, 7, 8]. Causes of ACHS include watershed infarction or ischemia, tumors such as glioma or neuroma, and viral encephalitis. It has been suggested, in most cases, that patients with normal lung function are able to normalize gas exchange during wakefulness by voluntarily increasing minute ventilation. However, during sleep, this voluntary response dissipates resulting in hypoventilation, hypercapnia, and hypoxemia [9]. In contrast to the CCHS, ACHS is sometimes reversible by addressing the underlying cause. When reversible, the management of ACHS includes temporary ventilatory support with noninvasive positive pressure ventilation and treating the underlying etiology. In one case report, a patient developed apneic episodes on a mechanical ventilator while sleeping and was found to have lesions in the bilateral lateral medulla of the brainstem that was thought to be related to patient's underlying multiple myeloma diagnosis. The bilateral medullary lesions improved with chemotherapy and the patient was successfully weaned off the mechanical ventilator to bilevel NIPPV [3].

Transient ACHS due to uncal herniation is rare and management strategies for the hypoventilation are lacking. In our case, the transient ACHS was successfully managed with bilevel NIPPV intermittently during wakefulness and continuously during sleep. Although rare, the clinician should be aware of the potential for recovery from ACHS after uncal herniation. In such cases, NIPPV can be considered to manage gas exchange abnormalities.

## 4. Conclusion

Given the paucity of literature, we aim to bring awareness of this life-threatening presentation of acquired hypoventilation syndrome and add to the clinical experience of favorable outcome when treated appropriately with both noninvasive ventilation support modality and the correction of underlying etiology.
